# Supplementary material for: Large-scale emergence of regional changes in year-to-year temperature variability by the end of the 21st century
Source: Nat Commun. 2021 Dec 13;12:7237. doi: 10.1038/s41467-021-27515-x (PMC8668997; doi:10.1038/s41467-021-27515-x)
Supplement: Supplementary file 1 — Supplementary Information [file 41467_2021_27515_MOESM1_ESM.pdf]

Supplementary information for: Large-scale emergence of regional changes in year-to-year temperature variability by the end of the 21st century

Dirk Olonscheck[1,2*], Andrew P. Schurer[1], Lucie Lücke[1] & Gabriele C. Hegerl[1]

[1] School of GeoSciences, University of Edinburgh, Edinburgh EH9 3JW, UK

[2] Max Planck Institute for Meteorology, 20146 Hamburg, Germany

[*] Corresponding author: dirk.olonscheck@ed.ac.uk

Content of this file

Supplementary Figures 1 to 8

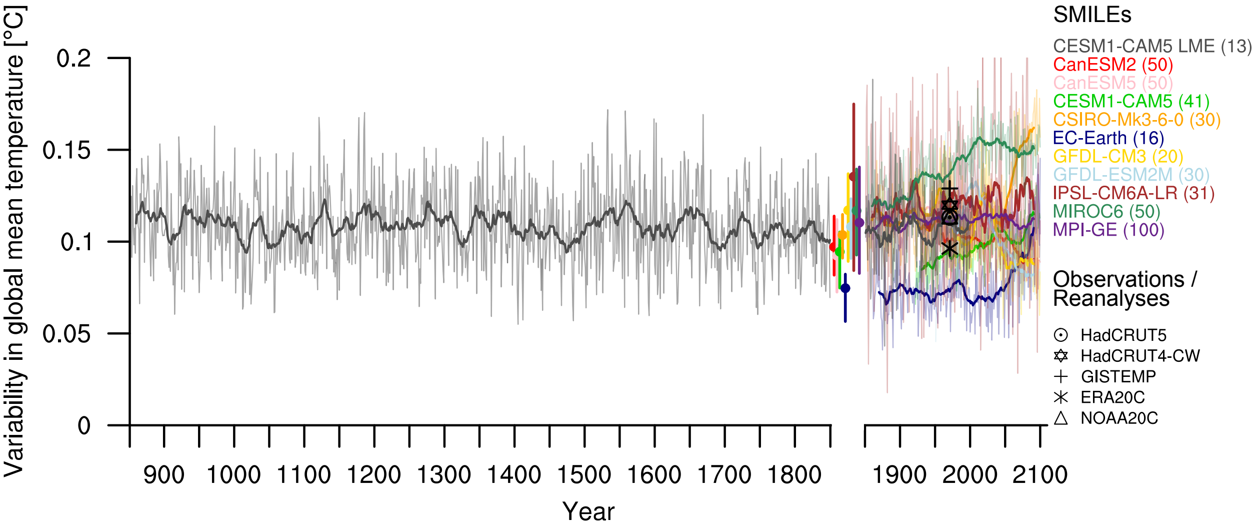

Supplementary Fig. 1: Evolution of interannual variability in global mean temperature from 850 to 2100 CE. Same as Fig. 1a, but for the interannual variability in global mean temperature.

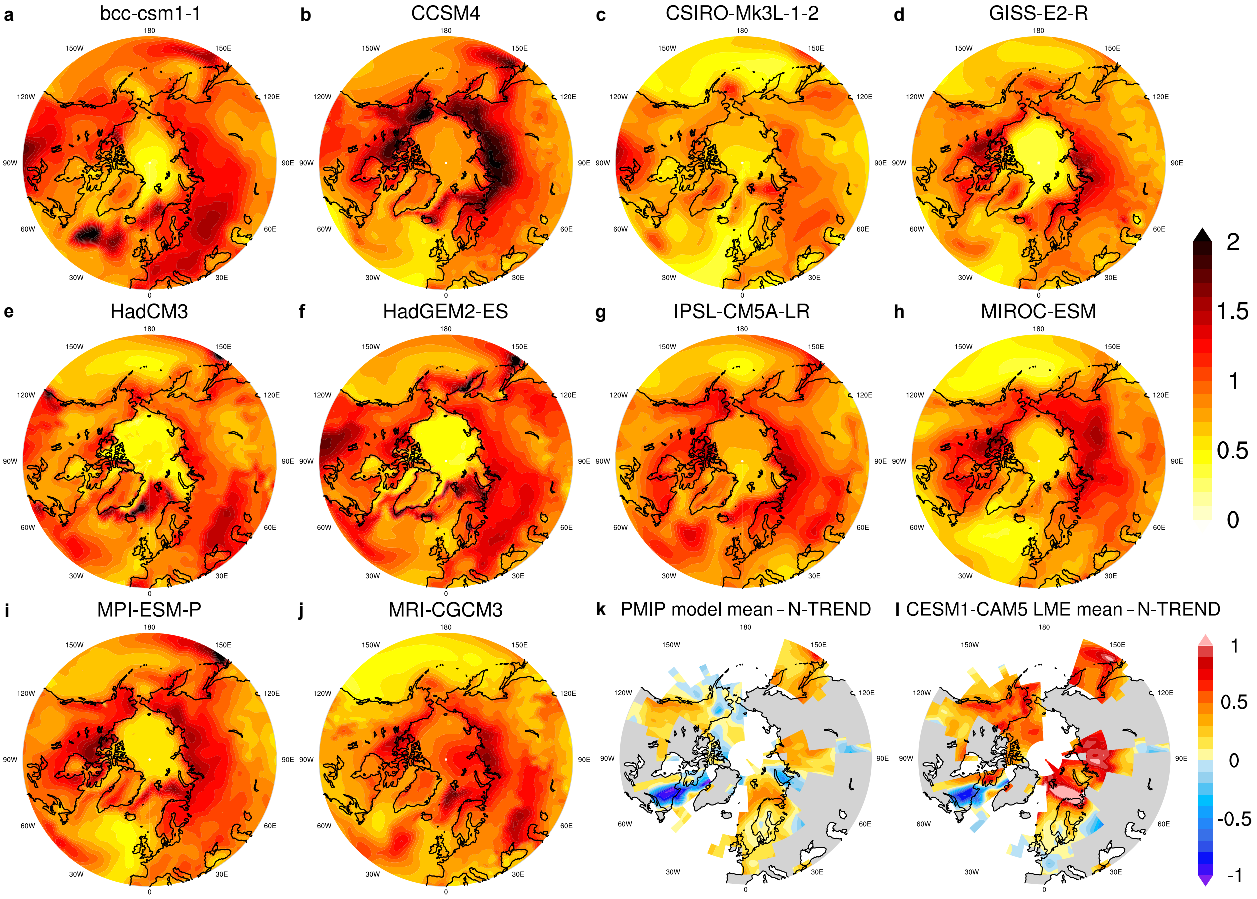

Supplementary Fig. 2: Simulated Northern Hemisphere last millennium temperature variability. a-j) Spatial pattern in summer (MJJA) from ten PMIP models. **k-l**) Difference in **k**) the PMIP model mean and N-TREND, and **l**) the CESM1-CAM5 LME mean and N-TREND. Note the different colour bar in **k-l**). Grey areas indicate land with no data. Compare with Fig. 2c-e.

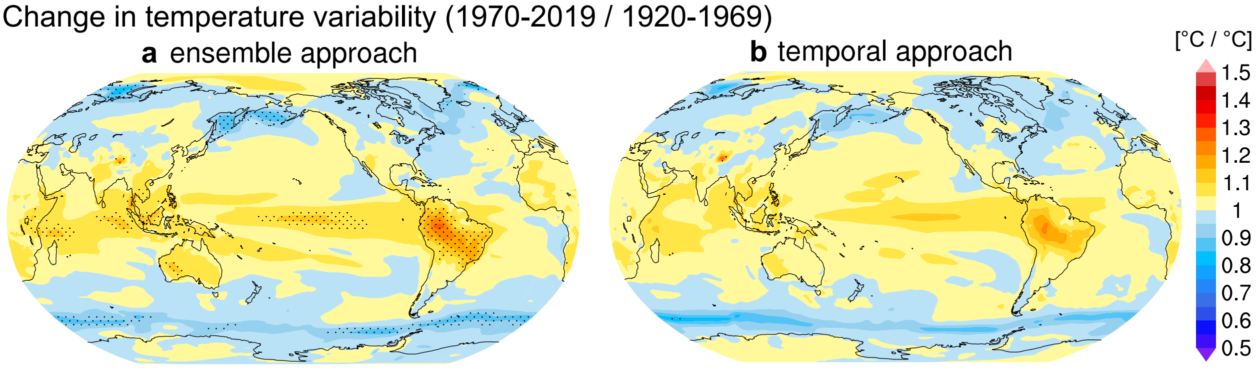

Supplementary Fig. 3: Comparison of the ensemble and temporal approach to quantify changes in temperature variability based on eight SMILEs. **a**) The ensemble approach quantifies variability with the ensemble standard deviation across ensemble members for each year, averaged across the full time period and all SMILEs (see Methods). **b**) The temporal approach quantifies variability with the temporal standard deviation across detrended 50-year periods for each ensemble member, averaged across ensemble members and all SMILEs. Each ensemble member is detrended by subtracting the ensemble mean of the respective model ensemble. The change in variability is determined as the ratio between the periods 1970–2019 and 1920–1969 in both approaches. Stippling marks significant changes at a 5% level based on an F-test. The ensemble approach shows larger regions of significant changes because of the larger sample size available for calculating the standard deviation.

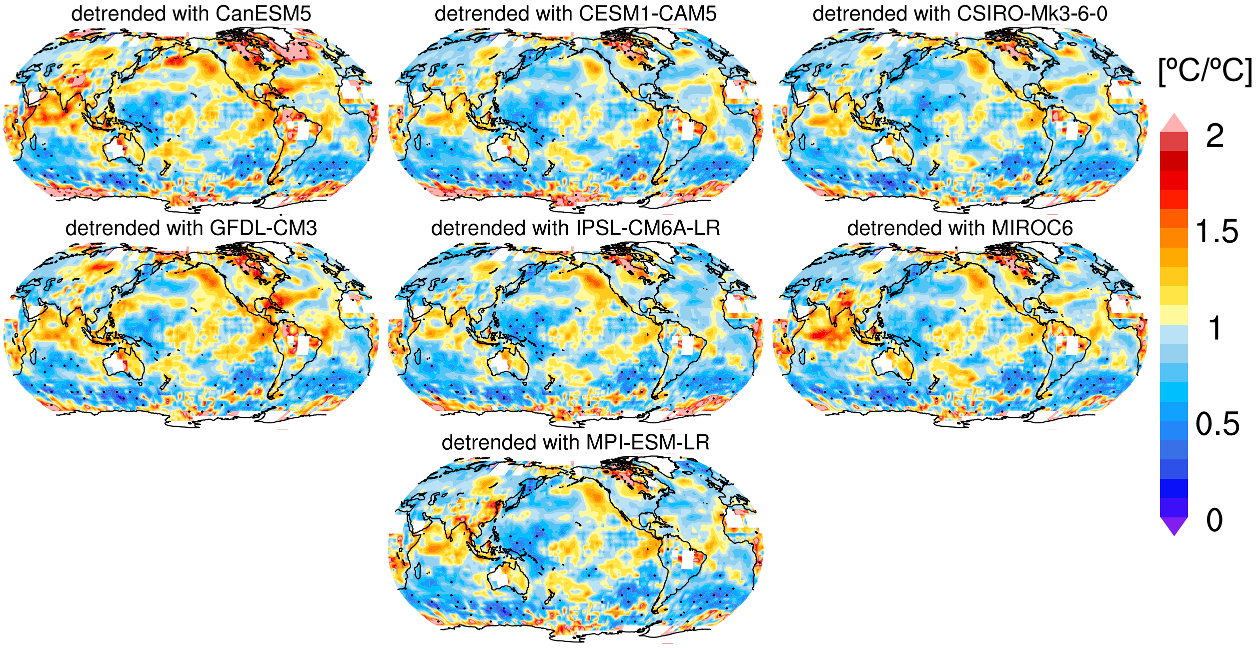

Supplementary Fig. 4: Dependency of observed changes of temperature variability on the detrending method. Changes in temperature variability between 1970–2019 and 1920–1969 for HadCRUT5 detrended with each a single-model ensemble mean instead of the mean of all single-model ensemble means used in Fig. 1c, Supplementary Fig. 5 and Supplementary Fig. 6. Stippling marks significant changes at a 5% level based on an F-test.

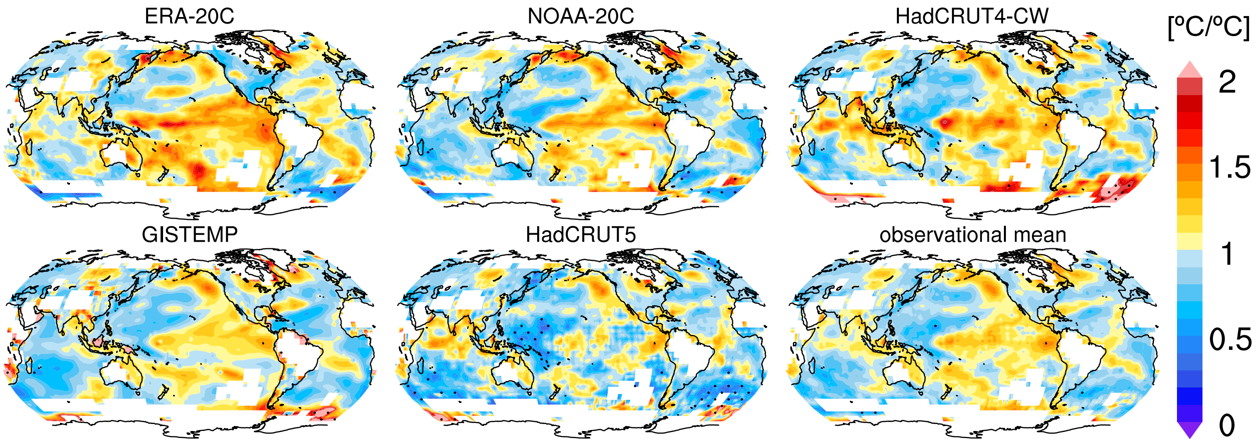

Supplementary Fig. 5: Consistency in changes of temperature variability between observational products. Changes in temperature variability between 1970–2019 and 1920–1969 for the five observational products ERA-20C, NOAA-20C, HadCRUT4-CW, GISTEMP and HadCRUT5, and the mean of the observational products. The observational products are detrended with the multi-model mean of the SMILE means, and the variability is calculated as temporal standard deviation over both 50-year periods. Grid points with missing or uncertain data in the HadCRUT5 dataset are masked out in all other products. Stippling marks significant changes at a 5% level based on an F-test. Note that the end year differs for the different observational products (ERA-20C: 2010, NOAA-20C: 2015, HadCRUT4-CW: 2018, GISTEMP: 2019, HadCRUT5: 2019).

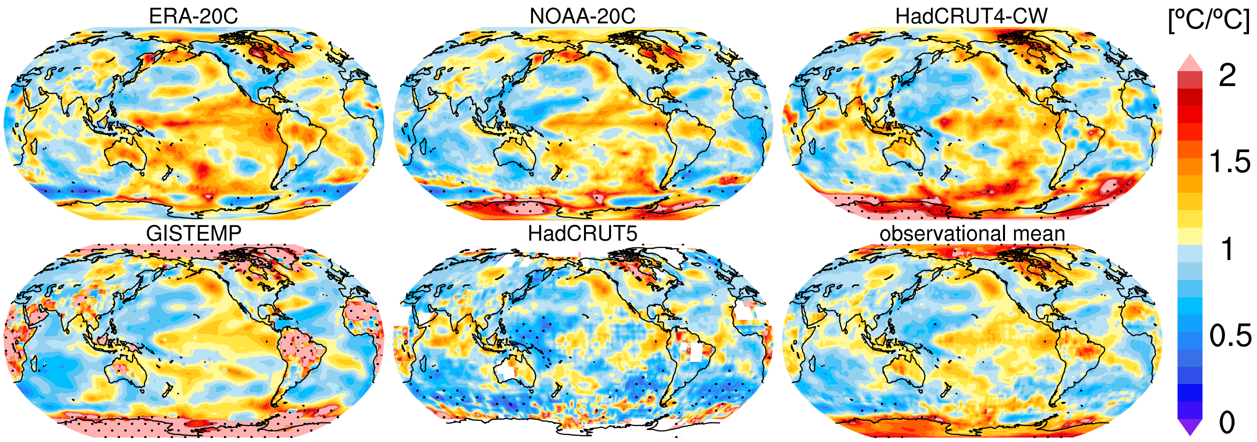

Supplementary Fig. 6: Consistency in changes of temperature variability between observational products. Same as Supplementary Fig. 5, but without masking. Stippling marks significant changes at a 5% level based on an F-test.

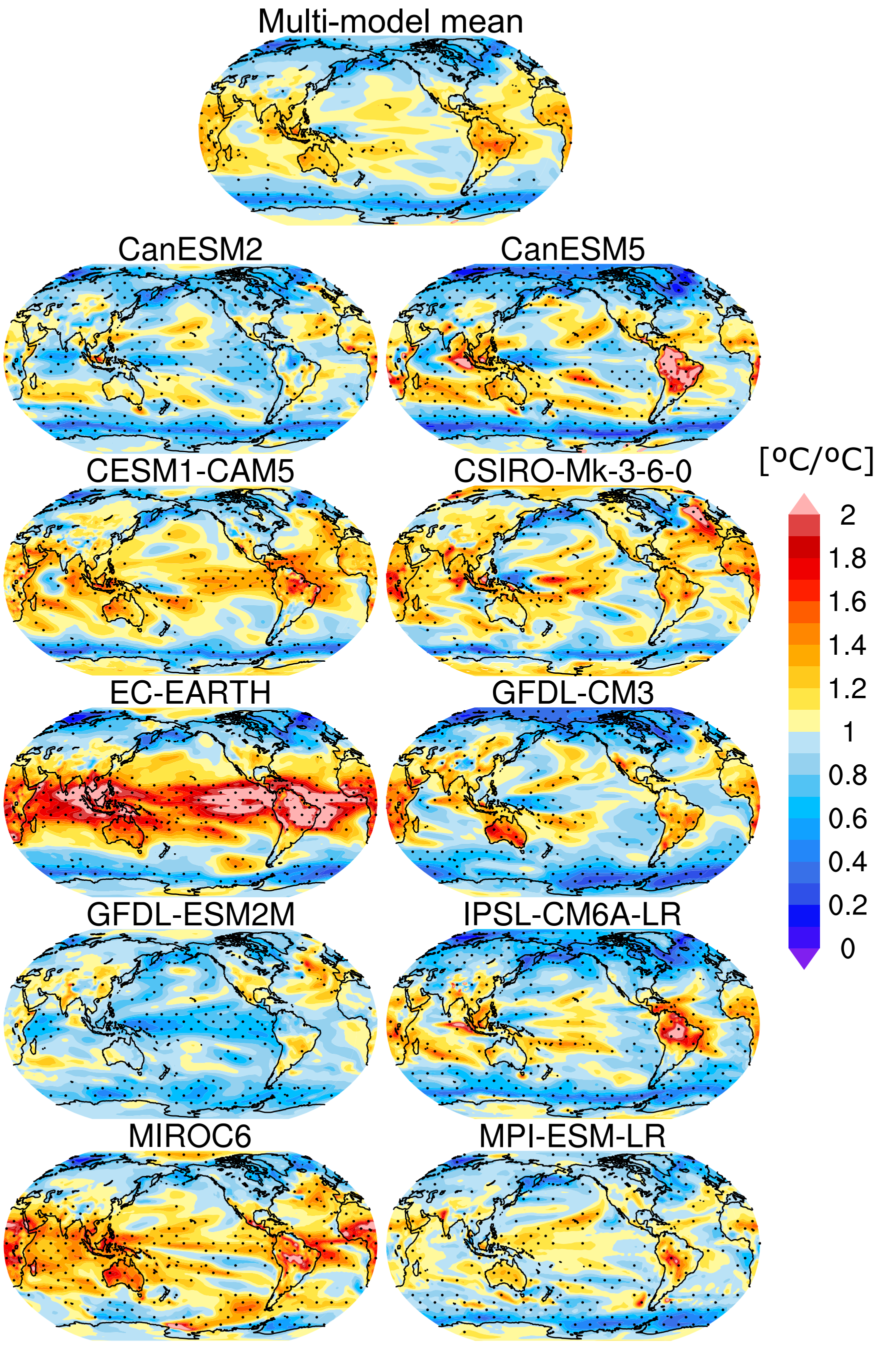

Supplementary Fig. 7: Projected changes in internal interannual temperature variability due to human influences. Distribution of temperature variability change for the 10-year average 2090–2099 with respect to the preindustrial variability from ten SMILEs forced with the emission scenarios SSP5-8.5 or RCP8.5. Stippling marks significant changes at a 5% level based on an F-test. Same as Figure 3f for the multi-model mean.

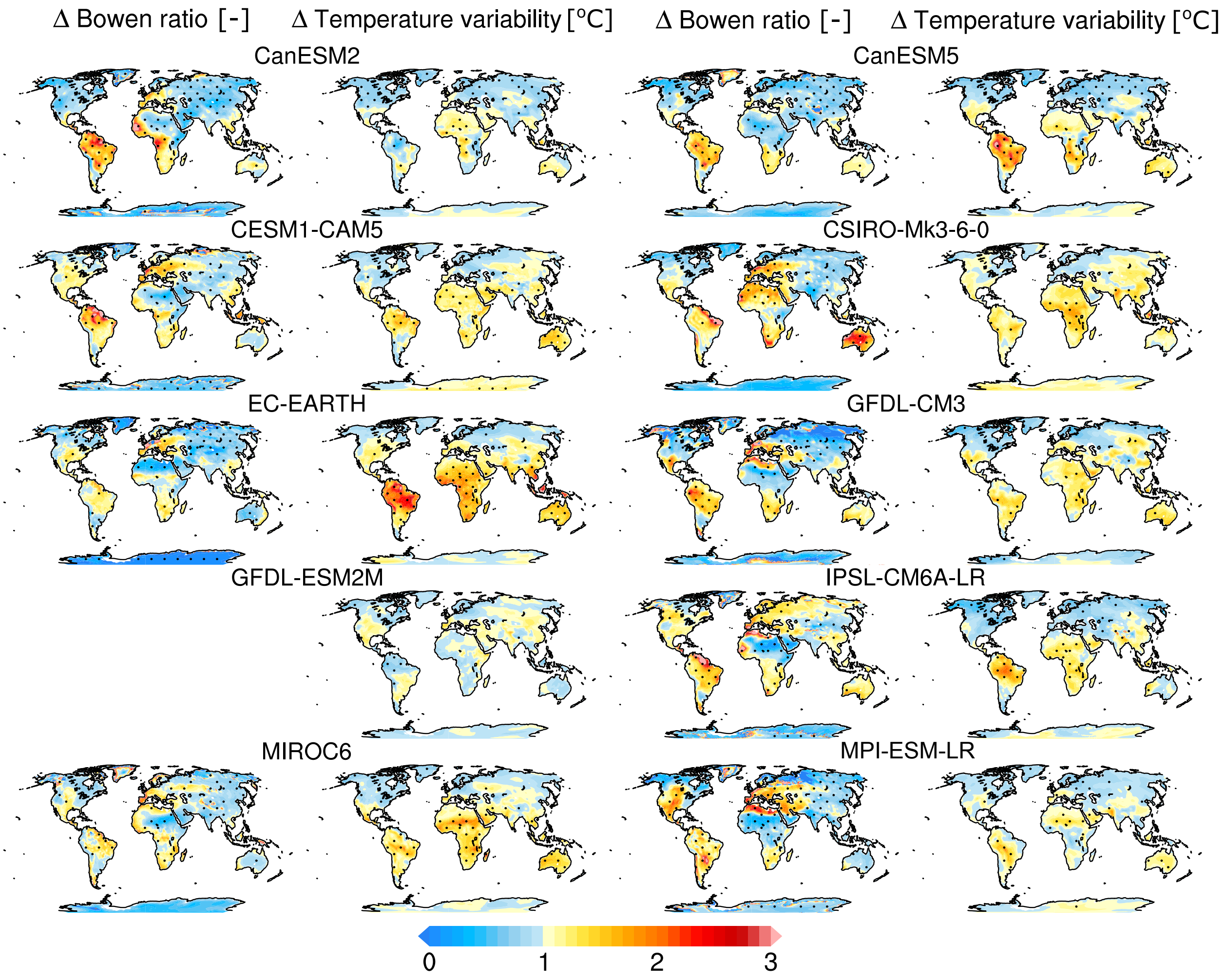

Supplementary Fig. 8: Comparison of changes in Bowen ratio to that in temperature variability in SMILEs. Ratio of changes in the Bowen ratio (left) and changes in the ratio of temperature variability over land (right) between 2090–2099 and 1950–1959 for all SMILEs individually. Stippling marks significant changes at a 5% level based on an F-test. Note that for EC-EARTH the Bowen ratio is based on four ensemble members, and for IPSL-CM6A-LR on six ensemble members only. For GFDL-ESM2M, data for calculating the Bowen ratio is missing.
